# Case report: Adrenal myelolipoma resected by laparoscopic surgery

**DOI:** 10.3389/fonc.2022.1058211

**Published:** 2022-12-05

**Authors:** Qingbo Feng, Hancong Li, Xinyang Chen, Xuping Feng, Jiaxin Li

**Affiliations:** ^1^ Department of Liver Surgery and Liver Transplantation Centre, West China Hospital, Sichuan University, Chengdu, Sichuan, China; ^2^ West China School of Medicine, West China Hospital, Sichuan University, Chengdu, Sichuan, China; ^3^ Department of General Surgery, Dafang County People’s Hospital, Bijie, Guizhou, China

**Keywords:** adrenal myelolipoma, laparoscopic surgery, case report, literature review, adrenal incidentaloma

## Abstract

**Introduction:**

Adrenal myelolipomas are benign tumors composed mainly of lipomatous elements with myeloid cells. With the development of medical imaging technology, the detection rate has gradually increased. We report a case of adrenal myelolipoma successfully excised through the laparoscope and reviewed existing literature in recent ten years to summarize the feasibility of the laparoscopic approach for this tumor.

**Case presentation:**

Herein, we described a case of adrenal myelolipoma resected by laparoscope in a 63-year-old male patient. He did not have any other symptoms except the incidental finding of a left adrenal mass. An abdominal CT examination revealed a mixed-density lesion containing some amount of adipose tissue. In conjunction with the patient’s willingness, we performed a laparoscopic operation to remove the lump. The definite diagnosis was confirmed as an adrenal myelolipoma according to the pathology. The patient recovered well postoperatively and without signs of recurrence at a 5-month follow-up.

**Conclusion:**

Adrenal myelolipoma is commonly benign, asymptomatic, and hormonal inactivity. A surgical strategy is suggested for high-complication-risk patients. The laparoscopic approach is safe and effective with an obvious advantage over open procedures.

## Introduction

Adrenal myelolipomas (AMLs) are rare, benign, mesenchymal neoplasms, consisting of mature adipose mixed with myeloid elements, which were initially described by Gierke in 1905 (1). The incidence of AML is 3.3% to 3.6% of all adrenal tumors in the general population, which has reached the second most common adrenal incidentalomas (1). Although prevailingly asymptomatic, enormous AML can present unregulated pain in the abdominal or flank due to compressing the surrounding tissue, further associated with hemorrhage or rupture (2). Imaging studies contribute to the diagnosis and detectable rate of AML; pathological examination reaches a definitive diagnosis (3). Generally, conservative treatment strategies are preferred. However, if symptoms develop, mass growth accelerates rapidly, or if it exceeds 6 cm, adrenalectomy will be the best option (4). While open surgical removal of giant AMLs is considered to be the treatment of choice, there have been increasing reports of giant AMLs successfully managed with minimally invasive techniques. Herein, we describe a case of AML and conduct a literature review on laparoscopic resection of AML in the last decade, emphasizing the feasibility of removing this kind of tumor by laparoscopic approaches.

## Case presentation

Our manuscript reporting adheres to CAse REport (CARE) guidelines (5).

We present a case of a 63-year-old male patient with no clinical symptoms but revealed a left adrenal mass in a CT scan for a regular physical checkup.

**Figure 1:**
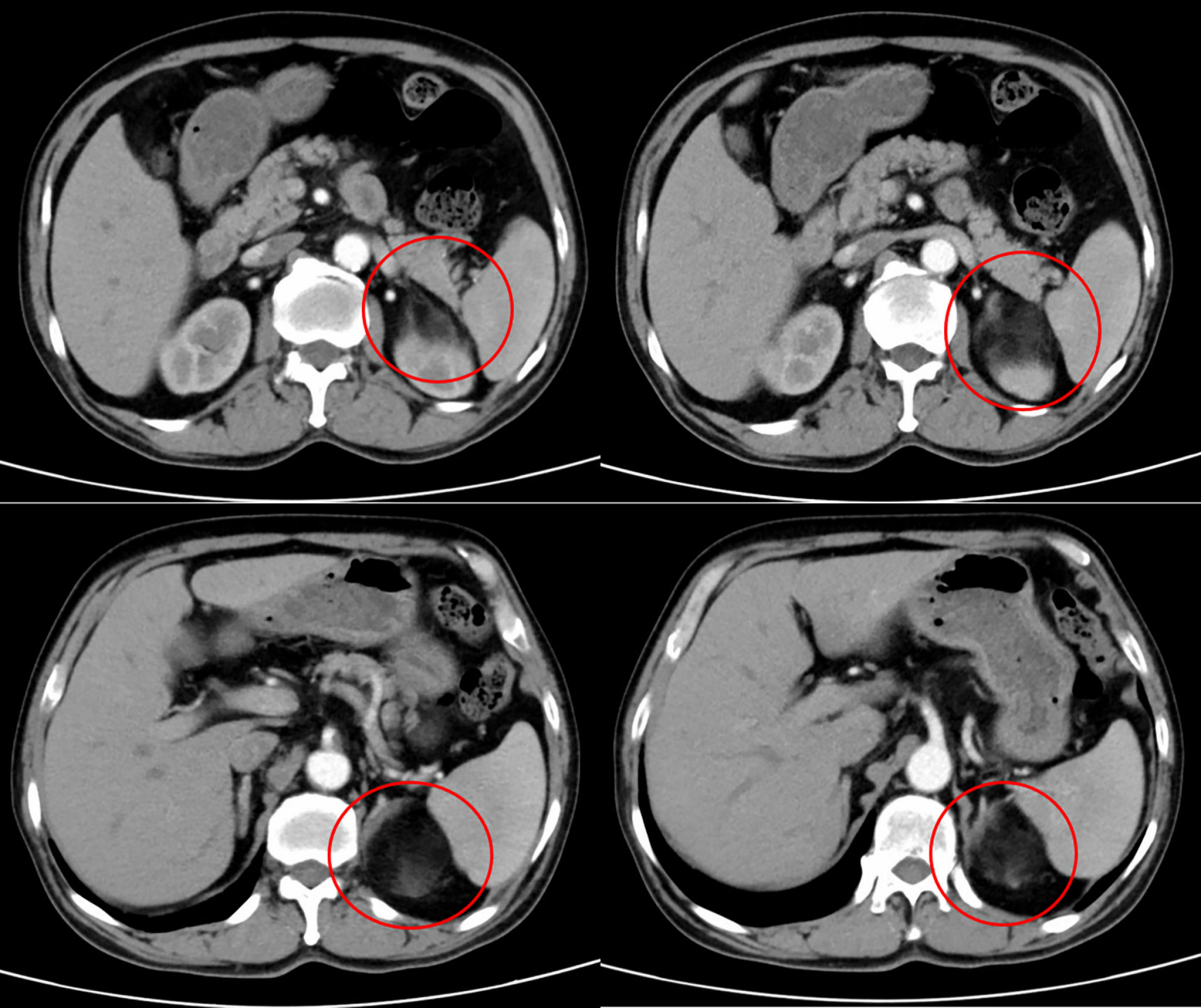
Abdominal computed tomography imaging, representing a left adrenal mass of 5.1×5.1cm.

His past history included II diabetes mellitus treated with gliclazide, and he had been controlled with propranolol and amlodipine for hypertension. Upon admission, he had good general condition and stable vital signs, without palpable abdominal mass. Laboratory measurements demonstrated elevated blood Epinephrine (0.71 nmol/L, normal values <0.34 nmol/L). The serum levels of the tumor biomarker as well as thyroid hormones were normal. CT-scan showed a left adrenal mass of 5.1×5.1cm. The lesion manifested as a mixed-density shadow that contain certain amounts of adipose tissue (**Figure 1**). Based on imaging features, we highly suspected that was an adrenal myelolipoma. Even though the patient was asymptomatic and the tumor size was moderate, we planned to proceed with surgery combined with his wishes. On 29-04-2022, the patient underwent a left-sided laparoscopic procedure at our department.

During the operation, the patient was placed in the supine and parted-legs position. Using Veress needles, a 1-cm transverse incision near the right navel edge was made to establish pneumoperitoneum with a constant pressure of 13-14 mm Hg. Five trocars were used: a 5-mm trocar was inserted under the xiphoid process. A 12-mm trocar was placed below the left costal margin in the midclavicular line, and the other two 5-mm trocars were placed in the midaxillary and anterior axillary lines. The fifth trocar (12 mm) was installed a little to the left of the navel as a laparoscopic observation hole. The resection was carried out with an ultrasonic surgical aspirator (CUSA; Cavitron Laser-sonic Corp., Stamford, Connecticut, USA), harmonic scalpel (Ethicon Endo-Surgery, Inc., Blue Ash, Cincinnati, OH, USA), and a bipolar clamp coagulation system (ERBE, Tubingen, Germany). Intraoperative ultrasound sonography (IOUS) was performed for exploration and localization. The posterior peritoneum was gradually opened along the lower margin of the pancreatic body, and a 5×5cm neoplasm was observed behind the pancreatic body, demarcated clearly with the left kidney and spleen. The blood vessels around the left adrenal gland were separated and clipped, then severed with an ultrasound knife. The tumor was completely removed and the resection specimen was collected in a plastic bag and removed *via* a 6-cm incision on the low abdomen. A plasma drainage tube was placed to monitor the left retroperitoneal cavity drainage concerning volume, content, and color. Operating time was 270 min and blood loss was 50ml. However, free gas within the left pleural cavity was revealed in the immediate postoperative color-Doppler ultrasound examination. Pneumothorax was suspected after a CT scan of the left lung. Closed thoracic drainages were initiated following instant thoracic surgical consultation. Thoracentesis was smooth and the patient was transferred to the PACU (post-anesthesia care unit) for resuscitation. The postoperative course was uneventful and the patient was discharged after 4 days. He was satisfied with the operative outcome and complied with the follow-up recommendations. Neither dysfunction nor tumor recurrence was observed so far.

**Figure 2:**
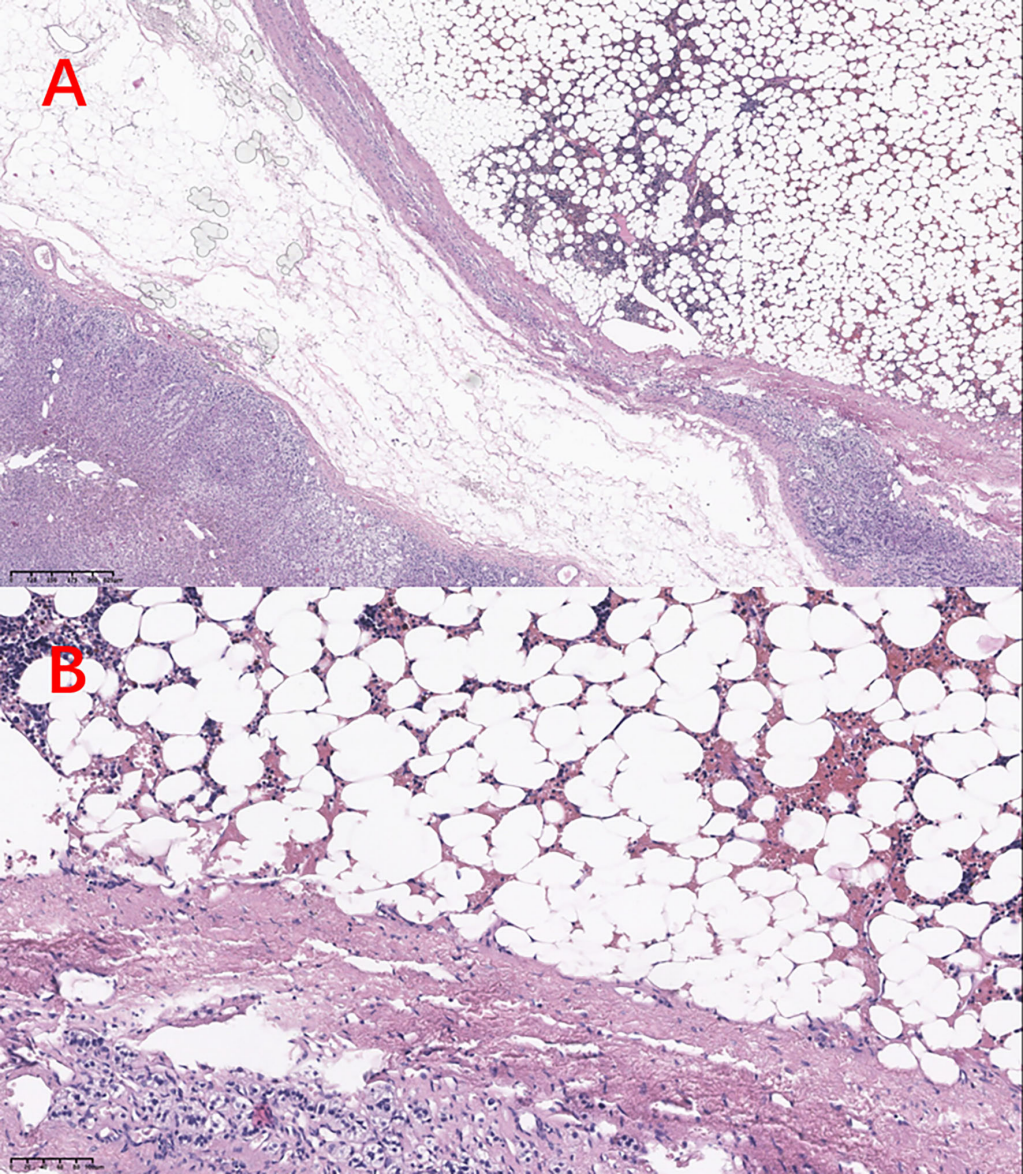
HE stains in **(A)** 10× view and **(B)** 40× view.

Grossly, a 5×5×4cm left adrenal tumor, in which, some necrosis and tumor-like changes were observed. Histopathology revealed a mixture of normal adrenal cells, adipose tissue, and three major hematopoietic components: myeloid, erythroid, and megakaryocytic lines (**Figure 2**). These findings confirmed the diagnosis of adrenal myelolipoma.

## Discussion

Adrenal lipomatous tumors are hormonally inactive and are often benign, myelolipoma is the most common type of them (6). Initially, it was mostly diagnosed as a postmortem finding. The prevalence at autopsy was estimated to be 0.08–0.4% in an autopsy series conducted in 1973 (7, 8). However, due to the widespread use of imaging techniques and especially high-resolution imaging procedures, incidental adrenal myelolipoma is increasingly reported. A clear explanation of adrenal myelolipoma etiology has not been determined. Several known risk factors contribute to its pathogenesis, including inflammation, degeneration, trauma, stress, obesity, hypertension, diabetes, and Cushing’s syndrome (9, 10). Reticuloendothelial cell metaplasia of adrenal capillaries, emboli from bone marrow, and adrenal embryonic remnants of hematopoietic elements have been suggested as postulated mechanisms (3).

Most AMLs occur unilaterally (more frequent on the right adrenal), but the extra-adrenal localization, such as the presacral area, spleen, stomach, thoracic, retroperitoneal, pelvic, renal, hepatic, and osseous locations are also documented (11, 12). The sexes are affected equally, primarily during their 5th and 7th decades of life (13–15). The majority of AMLs are asymptomatic and hormonally inactive (10). Upon initial diagnosis, the myelolipoma typically with a median size of 2–2.5 cm (usually <4 cm), in diameter (1, 14, 16). Tumors of a larger size may be palpable or may cause symptoms as a result of mass effect, even complicated by spontaneous bleeding and pain, which ultimately lead to hemodynamic shock (17). While AMLs do not generally synthesize hormones, they might sometimes coexist with primary aldosteronism, congenital adrenal hyperplasia (CAH), phaeochromocytoma, adrenal adenoma, and Cushing’s syndrome, creating excessive adrenal hormone levels (18).

The diagnosis of AMLs can be reliable by imaging techniques. A CT scan shows low-density fat (-10 to -30 Hounsfield units) mixed with higher-density marrow, which is a useful feature in identifying myelolipoma. In addition, areas of hemorrhage and calcification sometimes are found within the tumor (19). While MRI is more sensitive to detecting the macroscopic fatty tissue components, which usually hyperintense on the T1-weighted images and heterogeneously hyperintense on the T2-weighted images (20). On imaging, although infrequently, it needs to be differentiated from adrenocortical carcinomas with macroscopic fat, retroperitoneal liposarcoma, adrenal lipomas, teratomas, and angiomyolipomas (2, 21–24).

Conservative management by surveillance with regular imaging follow-up is the therapy of choice for small, and asymptomatic lesions (4, 25). Adrenalectomy is indicated when symptomatology ensues, size greater than 4-7 cm, at a high risk of rupture and bleeding, and suspicion of malignancy in an imaging study (4, 9, 10). As for AMLs >10cm, open surgery is recommended, while just several reports of tumors being removed using minimally invasive strategies. The laparoscopic operation was once considered contraindicated for adrenal tumors exceeding 5-6 cm (26). However, the laparoscopic approach is being increasingly used and extended for larger-size adrenal tumors, and we found 18 articles published reported 23 cases in recent 10 years (3, 4, 17, 25, 27–40). **Table 1** provides further details of these reports. Our literature review observes that AML with diameters up to 16 cm can be safely removed through transperitoneal laparoscopy (3, 4). Only one patient converted to open exploratory laparotomy due to multiple adhesions of the previous abdominal surgery which was conducted 6 years ago for a motor vehicle accident (27). Retroperitoneoscopic excision has also been reported as an option, though in limited case quantities (40, 41). Our literature review supports that laparoscopy is feasible and should be suggested as the procedure of choice concerning post-operative comfort, recovery time, scars, post-operative pain, and duration of hospitalization.

**Table 1:** A summary of reported adrenal myelolipoma resected by laparoscopic surgery in the last decade, arranged by published year.

First Author	Year	Country	Size (cm)	Sex	Age	Location	Operation approach	Chief complaint	converted to open	Operative time	Blood loss volume	Hospital stays
*Current study*	2022	China	5×5×4	M	63	left adrenal	transperitoneal laparoscopic	asymptomatic	no	270min	50ml	4
Tinozzi FP	2022	Italy	16 × 12 × 6	M	61	right adrenal	transperitoneal laparoscopic	right hypochondrium pain	no	160min	NR	5
Zulia YS	2021	USA	15	M	50	right adrenal	transperitoneal laparoscopic	right flank pain	no	NR	NR	NR
Kim DS	2021	Korea	9	M	50	right adrenal	transperitoneal laparoscopic	right flank pain	no	NR	NR	NR
Katsimantas A	2020	Greece	16.5 × 15 × 6.5	F	66	right adrenal	transperitoneal laparoscopic	asymptomatic	no	146min	NR	3
Introini C	2020	Italy	9×6	M	47	right adrenal	transperitoneal laparoscopic	back pain	no	NR	NR	5
Alkhalifa AM	2020	Arabia	6×4×6	F	46	right adrenal	transperitoneal laparoscopic	abdominal pain	no	NR	NR	2
Alkhalifa AM	2020	Arabia	6×5	F	35	right adrenal	transperitoneal laparoscopic	asymptomatic	no	NR	NR	3
Alkhalifa AM	2020	Arabia	9 × 7 × 6.6	M	45	right adrenal	transperitoneal laparoscopic	right flank pain	yes	NR	NR	2
Alkhalifa AM	2020	Arabia	6 × 5 × 4.5	F	47	left adrenal	transperitoneal laparoscopic	left flank pain	no	NR	NR	2
Yamamoto T	2019	Japan	14.3	F	69	left adrenal	transperitoneal laparoscopic	asymptomatic	no	NR	NR	NR
Piskinpasa H	2019	Bangladesh	Right:8×7×5;Left:4.1×2.3	M	41	bilateral adrenal	transperitoneal laparoscopic	asymptomatic	no	NR	NR	NR
Mhammedi WA	2019	Morocco	8.5× 8.5× 4.5	F	20	right adrenal	transperitoneal laparoscopic	abdominal pain	no	NR	NR	NR
Dotto RS	2019	Brazil	7.0×6.0×8.4	F	28	ectopic adrenal	transperitoneal laparoscopic	amenorrhea	no	270min	25ml	2
Liu N	2018	China	13.5×10.5×6.5	F	26	left adrenal	transperitoneal laparoscopic	secondary amenorrhea	no	NR	NR	NR
Molnar C	2017	Romania	4×5	F	65	right adrenal	transperitoneal laparoscopic	right flank pain	no	120min	NR	4
Soveid M Md	2016	Iran	6.5	F	26	left adrenal	transperitoneal laparoscopic	left flank pain	no	NR	NR	NR
Chaudhary R	2016	India	15×11	M	55	left adrenal	transperitoneal laparoscopic	left upper abdomen pain	no	210min	50-60ml	3
Yang Y	2015	China	4×4×3.3	M	40	right adrenal	transperitoneal laparoscopic	asymptomatic	no	NR	NR	NR
Park BH	2015	Korea	9.0×8.5	F	45	right adrenal	transperitoneal laparoscopic	right flank pain	no	110min	50ml	NR
Yamashita S	2014	Japan	4.5	M	49	right adrenal	transperitoneal laparoscopic	asymptomatic	no	117min	20ml	4
Yamashita S	2014	Japan	5	F	40	right adrenal	transperitoneal laparoscopic	asymptomatic	no	188min	160ml	4
Yamashita S	2014	Japan	3.5	M	45	right adrenal	transperitoneal laparoscopic	asymptomatic	no	152min	50ml	4
Wu ZS	2013	Taiwan	3.6	M	30	right adrenal	posterior retroperitoneoscopic	asymptomatic	no	160min	NR	2

NR: Not Reported.

As a minimally invasive approach, laparoscopic surgery has well-known advantages over open procedures, including reduced postoperative complications, better cosmetic results, and shorter recovery time. Surgical site infection (SSI) is one of the common postoperative complications, constituting critical damage to surgery patients (42). While laparoscopic surgery shows better performance in decreasing the incidence of SSI due to the smaller incision, less blood loss, and fewer drainage times (43–45).

In addition, a small incision in the minimally invasive procedure can relieve postoperative pain and minimize scarring (46). Also, the time to first flatus, early oral diet restoration, mobilization, and length of hospital stay favor the minimally invasive operation (44). Therefore, more conducive to the rapid rehabilitation of patients after surgery compared with the open approach.

Although limited cases were reported, retroperitoneal routes could also be utilized by the surgeon. Retroperitoneal laparoscopic adrenalectomy, which provides more direct access to the adrenal gland, approaches the lesion from the back without cutting the peritoneum (47). The safety of retroperitoneal laparoscopy has been confirmed by several meta-analyses (47, 48). Despite the disadvantage of the limited operative space, the retroperitoneal approach is now widely accepted as a fast and safe operation, with the merit of shorter surgical time, less post-operative pain, reduced complication rate, and faster recovery (49, 50). Direct access to the adrenal gland, avoiding intraperitoneal organic injury, may probably be the reason for these advantages.

Notably, our patient suffered from pneumothorax immediately after the procedure. The injury to the diaphragm and pleura caused by electrocautery during the dissection of the left adrenal gland tumor was speculated to be the cause of the left-sided pneumothorax. A similar condition secondary to laparoscopic adrenalectomy has previously been observed and reported elsewhere (51–53). Even though the incidence was not high, this experience reminded us to pay close attention to the gentle operation and be vigilant of complications. Since the injury wound is typically small, the pneumothorax can be resolved by suturing the tear under laparoscopy (52). In the present study, we performed a thoracocentesis after the abdominal cavity was closed. The patient recovered well without chest pain or dyspnea.

In addition, histological grading has important prognostic implications. Therefore, exact histological grading is critical for guiding the following management (54). In future research, we will strengthen cooperation with the Pathology department, conducting a large pathological review of the adrenal myelolipoma and consequently better individual treatment and prognosis.

To sum up, the detection of adrenal myelolipoma increased with the development of imaging modalities. However, insufficient awareness of this adrenal incidentaloma exists among clinicians. Studies for establishing a common guideline on the management of adrenal myelolipoma are needed. We suggest laparoscopy could be a safe and effective surgical approach for the treatment strategy.

## Data availability statement

The original contributions presented in the study are included in the article/**Supplementary Material**. Further inquiries can be directed to the corresponding author.

## Ethics statement

Written informed consent was obtained from the individual(s) for the publication of any potentially identifiable images or data included in this article.

## Author contributions

QF and HL drafted and revised the manuscript. XC and XF collected data and revised the manuscript. QF revised the manuscript for content. JL designed the study and revised the manuscript. All authors contributed to the article and approved the submitted version.

## Funding

This work was supported by Sichuan University from 0 to 1 project (No. 2022SCUH0017); Sichuan Science and Technology Plan Project “International cooperation in science and technology innovation/technological innovation cooperation in Hong Kong, Macao and Taiwan” (No. 2021YFH0095).

## Conflict of interest

The authors declare that the research was conducted in the absence of any commercial or financial relationships that could be construed as a potential conflict of interest.

## Publisher’s note

All claims expressed in this article are solely those of the authors and do not necessarily represent those of their affiliated organizations, or those of the publisher, the editors and the reviewers. Any product that may be evaluated in this article, or claim that may be made by its manufacturer, is not guaranteed or endorsed by the publisher.
