# Supplementary material for: Case report: Adrenal myelolipoma resected by laparoscopic surgery
Source: Front Oncol. 2022 Dec 5;12:1058211. doi: 10.3389/fonc.2022.1058211 (PMC9760819; doi:10.3389/fonc.2022.1058211)
Supplement: Supplementary file 1 [file DataSheet_1.pdf]

Case report: Adrenal myelolipoma resected by laparoscopic surgery

Qingbo Feng[1#], Hancong Li[2#], Xinyang Chen [1#], Xuping Feng[2], Jiaxin Li[1*]

[1] Department of Liver Surgery and Liver Transplantation Centre, West China Hospital, Sichuan University, Chengdu, Sichuan, CN 610041, P. R. China

[2]West China School of Medicine, West China Hospital, Sichuan University, Chengdu, 610041, Sichuan, CN 610041, P. R. China.

# These authors contributed equally to this work

*** Corresponding author:**

Jiaxin Li, Ph.D., MD, Department of Liver Surgery and Liver Transplantation Centre, West China Hospital, Sichuan University, Chengdu, Sichuan, CN 610041, People's Republic of China. **E-mail:** 407723080@qq.com

Supplementary Table S1. Care Guidelines Checklist

Topic	Item	Checklist item description	Location where item is reported
Title	1	The diagnosis or intervention of primary focus followed by the words “case report”	P1
Key Words	2	2 to 5 key words that identify diagnoses or interventions in this case report, including "case report"	P2
Abstract (no references)	3a	Introduction: What is unique about this case and what does it add to the scientific literature?	P1−2
	3b	Main symptoms and/or important clinical findings	P2
	3c	The main diagnoses, therapeutic interventions, and outcomes	P2
	3d	Conclusion—What is the main “take-away” lesson(s) from this case?	P2
Introduction	4	One or two paragraphs summarizing why this case is unique (**may include references**)	P3
Patient Information	5a	De-identified patient specific information.	P4
	5b	Primary concerns and symptoms of the patient.	P4
	5c	Medical, family, and psycho-social history including relevant genetic information	P4
	5d	Relevant past interventions with outcomes	P4
Clinical Findings	6	Describe significant physical examination (PE) and important clinical findings.	P4
Timeline	7	Historical and current information from this episode of care organized as a timeline	-
Diagnostic Assessment	8a	Diagnostic testing (such as PE, laboratory testing, imaging, surveys).	P4
	8b	Diagnostic challenges (such as access to testing, financial, or cultural)	-
	8c	Diagnosis (including other diagnoses considered)	P4
	8d	Prognosis (such as staging in oncology) where applicable	-
Therapeutic Intervention	9a	Types of therapeutic intervention (such as pharmacologic, surgical, preventive, self-care)	P4
	9b	Administration of therapeutic intervention (such as dosage, strength, duration)	P4-5
	9c	Changes in therapeutic intervention (with rationale)	-
Follow-up and Outcomes	10a	Clinician and patient-assessed outcomes (if available)	P6
	10b	Important follow-up diagnostic and other test results	P6
	10c	Intervention adherence and tolerability (How was this assessed?)	-
	10d	Adverse and unanticipated events	-
Discussion	11a	A scientific discussion of the strengths AND limitations associated with this case report	-
	11b	Discussion of the relevant medical literature **with references**.	P6-P11
	11c	The scientific rationale for any conclusions (including assessment of possible causes)	P6-P11
	11d	The primary “take-away” lessons of this case report (without references) in a one paragraph conclusion	P11
Patient Perspective	12	The patient should share their perspective in one to two paragraphs on the treatment(s) they received	-
Informed Consent	13	Did the patient give informed consent? Please provide if requested	**Yes** √☐ **No** ☐
